# An exploratory study of the policy process and early implementation of the free NHIS coverage for pregnant women in Ghana

**DOI:** 10.1186/1475-9276-12-16

**Published:** 2013-02-27

**Authors:** Sophie Witter, Bertha Garshong, Valéry Ridde

**Affiliations:** 1 FEMHealth project, Immpact, Institute of Applied Health Sciences, University of Aberdeen, Foresterhill, Aberdeen AB25 2ZD, UK; 2 Research and Development Division, Ghana Health Services, Accra, Ghana; 3 Department of Preventive and Social Medicine, Medical Faculty, University of Montréal, 3875, rue Saint-Urbain, Montréal QC, Canada; 4 Centre de recherche du Centre hospitalier de l’Université de Montréal, Montréal (CRCHUM), Montréal, Canada

**Keywords:** Maternal health, Exemptions, Ghana, Health insurance, Policy process, Implementation

## Abstract

**Background:**

Pregnant women were offered free access to health care through National Health Insurance (NHIS) membership in Ghana in 2008, in the latest phase of policy reforms to ensure universal access to maternal health care. During the same year, free membership was made available to all children (under-18). This article presents an exploratory qualitative analysis of how the policy of free maternal membership was developed and how it is being implemented.

**Methods:**

The study was based on a review of existing literature – grey and published – and on a key informant interviews (n = 13) carried out in March-June 2012. The key informants included representatives of the key stakeholders in the health system and public administration, largely at national level but also including two districts.

**Results:**

The introduction of the new policy for pregnant women was seen as primarily a political initiative, with limited stakeholder consultation. No costing was done prior to introduction, and no additional funds provided to the NHIS to support the policy after the first year. Guidelines had been issued but beyond collecting numbers of women registered, no additional monitoring and evaluation have yet been put in place to monitor its implementation. Awareness of the under-18 s policy amongst informants was so low that this component had to be removed from the final study. Initial barriers to access, such as pregnancy tests, were cited, but many appear to have been resolved now. Providers are concerned about the workload related to services and claims management but have benefited from increased financial resources. Users still face informal charges, and are reported to have responded differentially, with rises in antenatal care and in urban areas highlighted. Policy sustainability is linked to the survival of the NHIS as a whole.

**Conclusions:**

Ghana has to be congratulated for its persistence in trying to address financial barriers. However, many themes from previous evaluations of exemptions policies in Ghana have recurred in this study – particularly, the difficulties of getting timely reimbursement to facilities, of controlling charging of patients, and of reaching the poorest. This suggests that providing free care through a national health insurance system has not solved systemic weaknesses. The wider concerns about raising the quality of care, and ensuring that all supply-side and demand-side elements are in place to make the policy effective will also take a longer term and bigger commitment.

## Background

Ghana has been selected as a case study for this research as it has actively engaged in a number of policy reforms in recent years to increase the financial accessibility of maternal and child health care. In 2003–4, exemptions were introduced for delivery care, first in four regions and then in 2005 across the country. This policy was later superseded, in 2008, by free coverage of all pregnant women within the National Health Insurance Scheme (NHIS), which had started in 2005. While the first phase has been relatively thoroughly evaluated [1], there is less understanding about the recent NHIS reforms in terms of access to reproductive and child health services, which includes the decision in 2008 to extend free coverage to all under-18 s, regardless of parental membership. Understanding the implications of the recent financing reforms for access to quality reproductive and child health services is very policy-relevant in Ghana today, and for other countries in the region, particularly those which are introducing social health insurance programmes and exemptions from payment for whole population groups.

Ghana has made significant progress in health outcomes: infant mortality has reduced (from 77 in 1988 to 50 in 2008), while child mortality has reduced to 80 deaths per 1,000 births [2]. However, much targeted effort is required to ensure maternal health outcomes in particular are improved. Maternal mortality remains high, though it has declined in the past two decades from 740 per 100,000 live births in 1990 to 451 in 2008. Family planning acceptor rates have been climbing (from 25% in 2006 to 31% in 2009). Antenatal care coverage remains high at around 92% in 2009. Skilled deliveries have remained fairly constant, at around 45% in 2006–9 (though with annual fluctuations). Roughly half of women receive post-natal care (56% in 2009) [3]. Neonatal deaths account for 60 percent of the deaths in infancy. Despite progress, Ghana is still off track on both Millennium Development Goal (MDG) 4 and MDG 5 [4].

This study was designed to add to understanding of recent policies to reduce financial barriers to care for vulnerable groups in the region. The policy of providing free care under the NHIS for pregnant women was launched on the 1st July 2008. According to guidelines issued that month, the focus of the policy was on reducing maternal mortality [5]. It waived the NHIS premium and registration fees and waiting time for pregnant women and entitled them to the full package of care provided by the NHIS. It also covered the newborn for the first three months of their life. In relation to offering free membership to under-18 s, which was launched in September 2008, no specific guidelines were issued. These are relatively new policies, which have not been analysed publically to date. According the NHIS figures for 2010, more than 49% of its registered identity card holders were under-18, and 5% of them were pregnant women [6]. These groups therefore form a substantial part of the overall membership.

Previous studies, both in the region and in Ghana, of selective exemptions targeted at vulnerable population groups have shown that they are appreciated by the population and can increase utilisation of services and also reduce inequalities [1,7]. However, a number of weaknesses have also been noted, including commonly a lack of detailed planning for policy implementation, shortages of funding and other inputs in relation to the increased demand, poor communication of policies, poor specification of the exempted package, lags in payment (and/or longer term debts) in relation to providers, and increased strain on the provider-patient relationship [8-11]. In this study, we sought to understand whether an exemption approach embedded in a social insurance system had faced similar implementation difficulties or not. This is a question with resonance in the region and beyond. The push towards universal health coverage, combined with a focus on reaching the MDGs, has led a large number of countries to experiment with different ways of reducing financial barriers for pregnant women and children in particular. There is a growing interest not only in how different policies perform but also in how to improve their roll-out and implementation [12].

## Methods

The exploratory study was based on six main research questions, which were adapted from a checklist [13]. This checklist distilled good practices for implementation of exemptions policies, based on previous evaluations. The good practices centred on the six areas of policy design; the policy development process; dissemination of policy; resource allocation; payment systems; and management, monitoring and evaluation. The six questions aimed:

1. To understand the policy development process of the introduction of the national free maternal health policy and exemptions for children under 18 years.

2. To find out if the exemptions policy was costed and the process of resource allocation to implement it.

3. To find out how funds for the implementation of the policy arrived at the implementation level.

4. To find out if guidelines were put in place for the dissemination, implementation, monitoring and evaluation of the policy and how these were implemented.

5. To find out the perception of key stakeholders about the policy and its implementation.

6. To make recommendations to policy makers and managers for the effective implementation of the policy.

The study was conducted between March and June 2012, using two research methods – a literature review and key informant interviews.

A document review was conducted of relevant MoH and Ghana Health Service (GHS) policy documents on maternal exemptions policies in Ghana. These were gained through requests to the key informants and from the archives of the researchers. A search was also done through Google Scholar for published articles on maternal exemptions in Ghana. Search terms included Ghana and national health insurance or NHIS and pregnant women or children and free care or exemption. The few articles and documents of relevance were analysed thematically, using the six main research questions.

**Figure 1:**
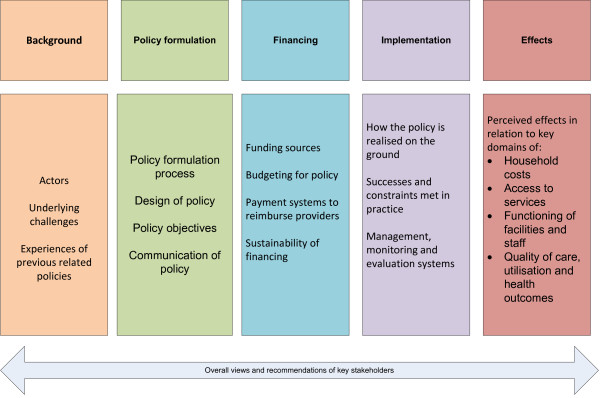
Thematic framework for results from exploratory interviews.

Thirteen key informant semi-structured interviews were conducted with a purposively selected sample comprising most of the main stakeholder groups, including parliamentarians [1], donors [1], staff from various concerned ministries (Health, Finance and Economic Development, and Women and Children) [5], health insurance representatives [1], and implementers within the GHS, at national [3] and district [2] levels. Participants were identified based on the posts they held, which included some responsibility for or involvement in the policy’s development and implementation. The two districts were selected pragmatically as being close to Accra – one urban and one rural. The interviews were tape recorded and transcribed. They were analysed thematically. The results are presented below following the framework presented in Figure 1, which adopts the logical sequence of policy development, implementation and effects.

Ethical clearance of the study protocol was given by the Ghana Health Service Ethical Review Committee in March 2012 (GHS-ERC: 18/01/12). Consent of all interviewees was sought. They were informed about the study and the purpose of the study, and that their participation was entirely voluntary. Confidentiality of all respondents was ensured. Given that the stakeholder group was limited, no identifiers are given for the citations below, to respect the anonymity of informants.

Although the scope of the original study covered both maternal and under-18 exemptions, the awareness of respondents of the details and operation of the policy for under-18 s were so limited that this component had to be removed in the analysis.

## Results

### Background to the policy

Most key informants pointed to concerns about statistics for maternal health and the need to reach the Millennium Development Goal 5 as the main driver behind the policy. The recognition that most women do not deliver with a skilled attendant was a major concern and the common perception was that removing financial barriers would facilitate access to health care service use by pregnant women. Many also raised other access barriers, including geographical, service availability, socio-cultural and informational barriers.

‘*Basically, it was designed to reduce maternal mortality by improving access to women who do not use maternal health services because of financial reasons’* (respondent 3)

Most respondents showed very little awareness of the lessons learned from the earlier phase of maternal delivery exemptions, which were still technically in force when the new NHIS-based policy came in, although the application had collapsed due to lack of funding [14]. Some referred to the earlier studies done by Immpact, but it was not clear whether these had influenced the new policy design.

### The process of developing the policy

According to informants, the President announced free care for pregnant women through the NHIS in 2008. The announcement followed a visit by the President to the UK in April where it was agreed that UK funds would be used to support the policy. The guidelines had to be prepared quickly as it was announced by the President for a fixed date. It was to start from the 1st July (Republic Day – a public holiday). Maternal mortality was declared a national emergency by the Minister of Health. After this, a maternal mortality conference was held in July 2008, with representatives from across Ghana and also internationally, although the report of the meeting fails to mention the policy [15]. A safe motherhood task force was also set up in September 2008.

The main stakeholders were the Ministry of Health (MoH), GHS, Christian Health Association of Ghana and the NHIS, who worked together on the guidelines. Policy guidelines were issued on the 27th July [5]. They specified eligibility (all pregnant women, and newborns for the first three months of their lives) and benefits package (all health services which are normally provided within the NHIS package falling within the year, starting from the presentation of the pregnant woman to a health facility). The Ministry of Women and Children had done some advocacy for free maternal care.

Some complained that there was not enough stakeholder consultation and that the details had not been well articulated. It was generally believed that policy comes from the top down, and is developed administratively, without adequately involving implementers – for this and other policies.

‘*We at the lower levels were not involved. As I said the stakeholder involvement was not good’* (respondent 6)

‘*It’s political expediency - the politicians say that I want it done now, so you have to do it’* (respondent 11)

### Communication of policy

Official stakeholders within the health services and the insurance scheme reported that they were informed about the policy through circulars, letters and memos. However, not all stakeholders were well informed. One of the key stakeholders, for example, was not aware that registration fees were waived for pregnant women. Not all the relevant departments in the MoH were able to recall receiving a written copy of the guidelines. Some stakeholders within the Ministry of Health were not fully aware of the details of earlier policies either. Respondents at district level also reported some lack of clarity on how to implement the policy due to different rules being applied.

‘*There was some correspondence from national level sort of that tried to state what it is and what it’s not but it was not widely disseminated, there were some confusions about it and when insurance took over, the insurance started making their own rules about who qualifies and that you need to come to the hospital and get a pregnancy test and things before you go to the insurance’* (respondent 6)

Funds for training staff in how to implement the policy were said to be lacking, so communication with the health system was restricted to sending the guidelines to districts. For the public, the media, Members of Parliament and District Chief Executives took up and passed on the message of the President.

### Budgeting & funding sources

Prior costing and budgeting of the policy was not done, however some estimates may have been made after the decision was taken, according to key informants. The Ministry of Finance allocates funds based on agreed annual priorities and work plans with the MoH but was not aware of any additional allocation to support this specific policy. Although the United Kingdom government supported the policy, the budget funding was already in place and the overall amount of aid was not increased, so in reality there was no new money for this policy, although a notional £4.5 million was allocated to the policy from the UK aid programme. In the first year, the MoH made a transfer to the NHIS to cover some of the costs. There continued to be some support to the implementation of the policy but it was not thought to be adequate.

‘*No cost analysis was done to find out if this 4.5million, how many years it will take, how many women it’ll cover, no’* (respondent 11)

‘*As up to today, nobody knows whether the money has been transferred to the health insurance or not’* (respondent 11)

There was a lack of understanding amongst these key informants of how the policy was being funded. It was assumed by many key informants to be funded separately by government (from external sources), whereas in reality the funding is internal to the NHIS, borne from its routine sources. Many informants believed that having a government budget line for the policy was important for its sustainability.

### Reimbursement systems

Services are repaid in a standard way for the NHIS, based on monthly claims. Reimbursement amounts are separated into service costs (based on agreed rates of fee per episode) and drugs, which are charged according to use. The fees per episode vary according to facility types, with higher level and private facilities paid at higher rates (reflecting their cost and subsidy structures) [16].

The policy implementation is affected by the funding delays (said to average 5 months), which are more generally an issue within the NHIS, and which cause facilities to withhold free services.

‘*If there are delays, it’s just the delays in payment of general health insurance claims and not on maternity alone*’ (respondent 11)

‘*My main concern is the arbitrariness with which the facilities can decide that this month I won’t do it because I haven’t been paid. These are some of the problems’* (respondent 3)

Some reported that reimbursements for maternal health care were handled more quickly than other general NHIS claims, but generally the process for vetting claims can be laborious and slow. Some regions within the NHIS system are currently piloting capitation, and this is reported to be causing additional problems with providers, who are resistant to this payment method.

### Sustainability

The policy was perceived as having been kick-started by funds from the UK government. That raised concerns amongst some stakeholders about what would happen when the one-off grant ended. A number of respondents recognised that there is no dedicated longer term funding for the policy.

‘*The policy is working, the only boring aspect of it is that, you know it’s for a period, when the funds are not there, how are we going to continue the policy*?’ (respondent 9)

Given that the policy is implemented within the NHIS system, most stakeholders consider it is sustainable if the health insurance is sustainable and if there is political commitment.

‘*It’s sustainable as much as we have the will to make it sustainable, and I can say that to the NHI as well - as long as Ghana decides that they want it to happen, it’ll happen’* (respondent 12)

However, others are sceptical about the something for nothing approach:

‘*It’s not sustainable. People should contribute and I think we have the platform*’ (respondent 6)

No analysis of financial impact has yet been conducted. However, in 2008 the fixed transfer to district schemes per exempted member of any type was 14 Ghanaian cedis (GhC). Overall figures for claims and expenditure were lacking to assess the adequacy of this subsidy, but in the case of pregnant women, the NHIS tariff for ANC, normal facility delivery and postnatal care at the lowest level of facility would cost just over GhC 14. Any additional complication, illness during pregnancy or seeking care at higher levels would therefore have been certain to push the cost over the subsidy level [16]. Analysis carried out by the NHIS itself indicated that a growing deficit would accrue to the NHIS from the scheme, in the absence of additional support [17].

### Policy implementation

Implementation of the policy has reportedly varied due to different interpretation of the policy by various implementers. Some of these generated additional barriers for women. For example, some requirements for eligibility were imposed:

‘*One day we heard that before you qualify, everybody must have a pregnancy test and then we had to go and complain that why a midwife should do a pregnancy test to tell you that this woman is pregnant, so there were certain problems. Some of them will demand a scan before you qualify for reimbursement … which was not free’* (respondent 3)

The card is given for a year and covers all care within that year (maternal and incidental, such as malaria), according to key informants. But there have been differences in local interpretation of the package of care: ‘*Some of them by the mere word of abortion, then they won’t pay but if the doctor wrote miscarriage then they will pay’* (respondent 3)

‘*The weaknesses are the misalignment between protocols where health insurance determine what they will pay irrespective of what the service is suppose to provide; we keep going back and f*orth’ (respondent 3)

Registration was also a barrier initially. In theory people have to register, have their photo taken, get a card – all of which takes time and money and can delay access to care. At the start, there were said to be disputes over whether women could be treated without these, and how to best organise the registration process for them.

‘*For example, if someone comes in Sunday, some of the insurance people say that if you treat them without being given authorisation, we won’t pay, so the person need to pay upfront*’ (respondent 6)

Some of the measures were linked to trying to combat perceived fraudulent claims.

‘*They say that the delivery side can be one of the weakest link that people can defraud them so they try to put in mechanisms to prevent them’* (respondent 6)

‘*Human beings we are like that, we always find ways from eating from the pot. So there were some mutual suspicions on both sides and sometimes people also felt that the insurance people are not paying them their due, which is sometimes also true because they’ll take away some the money for not writing something on it so they’ll deduct something’* (respondent 6)

Certain facilities were more reluctant than others to implement the policy e.g. the teaching hospitals, which are autonomous institutions.

‘*Officially, they will say they are included but unofficially they don’t give free maternal care’ (respondent 10)*

Some of these issues were however resolved over time (e.g. the NHIS was now reported to be paying for scans and allows palpation to confirm pregnancy).

‘*At all levels in the district, by them trying to work together, they improved their working so that it facilitated care for the women’* (respondent 6)

### Management, monitoring and evaluation

Within the MoH, the policy is supposed to be monitored by the Family Health Division, but there are some organisational problems, as the midwives who are implementing the policy fall under the Institutional Care Division (ICD). In reality, this division is said to be more concerned with managing doctors and nurses, so the midwives and this policy are to some extent falling between two stools.

*‘Under ICD or either safe motherhood, there is no champion midwife in either ICD or safe motherhood… maybe they need Director Midwifery Services so somebody who is the champion of midwifery services based in ICD’* (respondent 8)

The lack of champions of the policy, from national down to local level, was raised by some informants.

‘*There is no champion at the regional level, there is no champion at the district, it’s only the midwife at the facility level who is managing and the one who is supervising is the medical assistant, who half of the time is a male who has done no midwifery so he can’t talk about that’* (respondent 8)

Although the policy is monitored through routine NHIS systems, no plan was elaborated from the start to monitor or evaluate this policy specifically, although one is now planned, with UNICEF support, in 2012.

‘*It was part of the routine. It was integrated into the system*’ (respondent 6)

The Ministry of Women and Children are meant to represent the interests of women and children in relation to other ministries, feeding back information on priority areas and problems on the ground. However, they are not well resourced and have not been able to fulfil this role very effectively over the past few years. Technical departments are also not involved in monitoring the policy per se, though they do monitor changes to coverage which may result. The NHIS keeps records of numbers of women enrolled under the policy.

### Perceptions of the effects on household costs

There are various costs which are not covered by the policy, including transport and minor personal items which are required for a delivery, according to KI.

‘*The financial access issue has been sorted out but it still depends, it may not be covering everything. It will not cover for instance your transport cost to the hospital and back. It will not cover the food you’ll eat and so on and so forth and certain petty things. When you are going to deliver, you need to go with a pad and all those things; it is not covering those ones but the major things done for you are covered’* (respondent 7)

The NHIS has a list of approved drugs, so drugs which are off this list are charged, though these should not generally be necessary.

‘*They say it’s proprietary drug so you have to buy it and maybe they’ll say buy this or do this. I mean some of the facilities they’ll always find some excuse and take some cedis from you. In the ward, they’ll collect ward fees and they’ll ask you to bring some soap and dettol and some things’* (respondent 6)

There are also reports by informants of additional or informal charging, either opportunistic or related to delays in reimbursements by the NHIS. This is not specific to the free care policy but applies across all sorts of services. There are even cited instances of double-billing (to the patients and the NHIS).

‘*You get to a facility and you are entitled to some medications, they say we don’t have and they write it for you, what do you do? You have to go and buy’ *(respondent 11)

‘*Some of the answers we get from some of the facilities: if I have not received reimbursement and I’m running on my own resources, then sometimes some of the things, you have to write it for the client to go and buy’* (respondent 7)

The culture of giving gifts to midwives is also cited as a factor driving informal payments, which can come to a significant cost for women.

‘*They say it’s the custom of midwives that when you come and deliver, you’ll come and say thank you to the midwife… and they determine what they should bring to thank them, so they tell you they want this soap, they want this perfume, they want this piece of cloth, so in the end the money the woman spends in buying things to say thank you to the midwife is more than the fee she would have paid if she were being charged’* (respondent 8)

### Perception of the effects on access

Despite these concerns, there was a feeling that the policy has reduced delays in accessing care for women.

*‘The strength is that it provides access and one good thing was that even if you are not registered or on admission for complications or anything, the hospital initiates the process of getting you registered and so you don’t have to go and register before you come’ *(respondent 3)

‘*Breaking the financial barrier is a very big strength. Then we used to hear of children or mothers being detained because they couldn’t pay their fees, now there’s nothing like that’* (respondent 5)

However, some services seem to have increased more than others:

‘*Antenatal uptake has gone up very dramatically. Assisted delivery has not gone up that much and there are service provision reasons for this, and I believe one of the reasons is certain demands that are made on the women especially when they are going to deliver’* (respondent 12)

Policy makers acknowledged that there are other barriers (financial and non-financial) to the use of health care services beside payment at the point of care. Other access barriers include distance to facilities, socio-cultural barriers, and supply-side barriers, such as the lack of availability of critical equipments and drugs at the point of need, lack of availability of skilled staff and poor attitude of providers. Other policies are in place to improve the situation – e.g. the policy of placing midwives in each community – however, these are also facing implementation challenges.

Differences in physical access to facilities also means that better off women are able to benefit disproportionately, according to some key informants.

‘*One of the major weaknesses of that policy was that, it was supposed to be a pro- poor policy but it didn’t work out because of the way the facilities are sited. The facilities in our country are sited more skewed towards the urban that have money and so those who were benefitting from the free care were those who did not really need to benefit’* (respondent 6)

### Perception of the effects on facilities and staff

Facilities and staff were reported to have faced increasing workloads as a result of the policy, especially in urban areas.

‘*The initial problem was congestion. I think now people have come out with contingency measures and they have reorganised themselves to be able to deal with the increasing numbers, especially in the urban areas where there was serious increase’* (respondent 3)

‘*It did increase the workload tremendously. The antenatals were crazy’* (respondent 6)

For the private sector, it is also reported as having driven up business:

‘*The private, they are happy because it’s increased the numbers because the private used not to get many but now if they are accredited, they have those numbers’* (respondent 11)

Some comment on the inadequate resources for providers, though it is not clear whether there is any difference between this policy and others services refunded by the NHIS. (Facilities are now paid for all services using a fixed cost per episode, but with variable charging for permitted drugs, according to actual usage). The tertiary facilities are reported to be particularly unhappy with the tariffs. Others point to discrepancies in payments made to different facility types.

‘*From the provider side, they also complain about the tariffs they get from health insurance. They think it’s inadequate, health insurance also thinks that whatever we put in is also inadequate so for money, it’s never enough, we just have to manage and see that the policy is carried out’* (respondent 5)

However, it is also important to note that the increase in workload had meant a reported growth in revenue for providers, which was reported to have fed into some improvement in some of the facilities.

‘*The overall effect, because it was channelled through the insurance, capital influx in terms of IGF [*revenues from user fees and insurance payments*] increased for facilities’* (respondent 6)

However, at individual staff level, the incentives embodied within the policy may be less encouraging, leading to some evidence of ‘passive resistance’ from providers.

‘*A circular was issued to all midwives to stop collecting anything because insurance was going to pay for it but after that, in some areas, some midwives at the periphery will no longer take a labour case after eight pm’* (respondent 11)

### Perceived effects on quality of care, utilisation and health outcomes

Some key informants report concerns that the increase in workload has affected quality of care negatively. Some also mentioned that there were incentives not to refer, so as to keep the full reimbursement and cost cutting by providers.

‘*People were keeping patients to deliver to the end so that they also benefit or you pay for their bill before they leave, so those were some of the challenges’* (respondent 3)

‘*They don’t give the necessary medications they are supposed to do because they are doing more of capitation within the program because all these cases get a certain package*’ (respondent 3)

There are also some fears that the policy provides an incentive to increase fertility, and for women to make frivolous use of services, though equally others protest that both of these are not plausible. Most perceive an increase in supervised delivery rates, in caesareans in particular, and a reduction in maternal mortality.

### Overall views of the policy and stakeholders’ recommendations

Key informants were positive about the policy as a whole and about the need to address financial barriers – only one advocated for a major change, involving a shift to means-tested support.

*‘For the future, this policy should stay; the government should find a way to support it and all the players, everybody who has something to do with it should make sure it stays’* (respondent 2)

They made a variety of recommendations to strengthen and supplement it, including:

• Better public education, to increase demand for the service

• Improving access and transport, so that all can benefit, and in a timely way

• Some suggest conditional cash transfers to cover transport costs

• The more general cultural barriers also need to be addressed for the policy to meet its goals

• Complementary measures are needed on the supply side, including ensuring that there are enough midwives.

• Ensuring that the health system has the necessary supplies and facilities to provide the services

• There should be good general monitoring, but also focussed on equity

• In order to sustain and extend the policy, longer term funding sources need to be identified

• Family planning services should be added to the package, as this is cost effective and saves lives

## Discussion

The study was based on existing literature – grey and published – and on a limited number of key informant interviews. The key informants reflected key stakeholders on the health system and official side – users and civil society organisations were not represented as this was an initial exploratory study into policy process and implementation issues. The two districts were in the Greater Accra region, so the geographical scope was also small. The limitations of these methods have to be acknowledged. To a large extent, the study serves to raise questions for further research, rather than definitively answer them.

For the maternal exemptions, there is a clear understanding of the objectives of the policy, though few refer back to the experiences of predecessor policies (the delivery exemption policy and the exemption of under-fives, both still theoretically in place when these new initiatives came in). This may be a missed opportunity, as many of the themes of the interviews reflect problems identified before. This has also been raised in other countries in the region – for example, in Niger or Mali, NGO pilot projects were not taken into account in developing national policies [18], while in Senegal, experiences in the first phase regions were not evaluated before scaling up [9].

The policy of free NHIS membership for pregnant women appears to have been a political initiative, which was introduced rapidly and with limited stakeholder consultation, which again is a finding shared in many other countries which have recently introduced targeted exemptions [10]. It was supported by DFID and was believed by many to be externally financed, but in fact there were no additional resources at its disposal beyond existing budget support, and it appears that the NHIS has been funding it out of its internal resources, at least after the first year. Its sustainability is therefore bound up with that of the NHIS more broadly, and indeed adds to financial concerns already outlined in previous studies [16,19].

Communication of the policy was limited to bureaucratic transmission of guidelines, as it was the case in many countries in the region [10,20], and some media coverage, which is thought to have reached most people, at least for the pregnant women policy.

Some variation in interpretation and implementation was noted for the pregnant women. Some were initial problems which were reported to have been subsequently addressed, though perhaps not for the teaching hospitals, which were reported to be not complying with the policy.

The main concerns for providers are delays in relation to reimbursements and the workload which claims processing creates - a finding which fits with other recent studies from Ghana [21] and is limiting the effectiveness of similar policies in the region [22]. However, there is some qualitative evidence that facilities overall are benefiting from the increased revenues relating to greater maternal care use, as we have seen in other countries such as Burkina Faso [23,24]. The impact of the policy on quality of care is not clear at this stage, but some concerns were raised in relation to the pressures of workload and the incentives not to refer and to cut costs which are generated by the tariffs.

From the point of view of policy effectiveness, the greatest concern is the presence of informal charging, which appears to be both a response to the delays in funding (legitimate charging, one might call it, to fill gaps) [25] and rent seeking by staff. From a staff perspective, the policy has added to their workload while potentially reducing their ability to sell items to and receive gifts from patients. Their response – passive resistance, which passes a variety of costs on to patients – undermines the policy’s goals of increasing access. Some recent studies from Ghana also raise concerns that the insured are discriminated against because they do not bring in direct payments [26].

The respondents’ perceptions are that utilisation has increased, but especially for some services (antenatal in particular, and possibly also caesareans) and in some areas (urban), where women have better access. This raises equity concerns, but also concerns in relation to improving maternal health. As deaths are focussed amongst poorer women and more remote women, efforts must be made to reach these groups. The continuing existence of geographical and socio-cultural barriers, combined with continued informal charging, may limit the benefits for the group of women who are most in need, although a recent study in Brong Ahafo did find significant benefits for poorer women from the policy [7]. More research must be done in Ghana to provide evidence and support action for the poorest.

In line with the manner of introduction of the policy, it seems that there were no comprehensive estimates of the cost of the policy, or ongoing financial support for it. The main difference from the earlier Delivery Exemption Policy is that this absence of sustained funding has been masked by the general revenues of the NHIS, which have subsidised the policy. As respondents correctly identified, the funding of the policy is as sustainable as the NHIS and the political commitment made to it. It has to be recognised, however, that the policy adds to the financial risks faced by the NHIS, as currently managed. A recent report by the World Bank suggests that the NHIS has “serious structural and operational inefficiencies and is on a trajectory to go bankrupt” [19].

Previous studies have emphasised the lack of donor involvement in supporting exemption policies in the region [10]. In this case, a donor appeared to play a catalytic role in kick-starting the policy, but then played no further role in funding or providing technical support for it.

Overall, key informants are positive about the policy, focussing largely on the complementary measures which are needed on the demand- and supply-side, and also the need for longer term funding and effective monitoring of the policy. After four years of operation, there is a clear need to understand better the cost effectiveness of this policy. The lack of monitoring and evaluation plan has meant that nothing beyond coverage of women has been routinely collected. The need for policy ‘champions’ at different levels of the health system and more clearly delineated responsibilities for managing it also emerge from the interviews.

The study findings are consistent with previous studies in Ghana relating to exemption policies, particularly in relation to problems of delays in reimbursement, and lack of incentives to award full exemptions at the service provider level [1,25,27]. The main change is that the costs are now borne by the NHIS, which is currently able to provide a more reliable funding source. Many of the issues raised by the key informants are also reflected in early studies on the NHIS, which have also found, for example, that facilities are benefiting from increased cash flow, while being concerned about tariffs and claims management workload [16]. The constraints found in Ghana are also echoed in other countries in the region [12,28].

While Ghana has been active in developing policies to improve financial access to reproductive and child health care, other supply-side challenges remain, which have considerable potential to undermine the goal of reducing maternal mortality (even if women and babies reach facilities). A recent assessment of Emergency Obstetric and Neonatal Care in Ghana [29] highlighted a number of ongoing challenges, including gaps in infrastructure, transport, human resources (especially in relation to equal distribution and essential skills), equipment, blood and drugs. 58% of deliveries are currently supervised by a skilled birth attendant in Ghana (ranging from 29% in the Northern Region to 80% in Greater Accra). Yet only 21% occur in a facility with partial Basic Emergency Obstetric and Neonatal Care services or better. Met need for emergency obstetric care was estimated at 34% (but just 7% in the Northern Region). Nationally, 7% of deliveries are c-sections, but only 4% of deliveries are caesarean sections performed in a Comprehensive Emergency Obstetric and Neonatal Care facility.

While the study set out to understand the development and implementation of the free care policy for the under-18 s, this component had to be dropped due to low awareness by respondents. People were unable to say how it was developed, funded or implemented, nor what its effects have been. As this policy bucks the regional trend of focussing exemption policies on mothers and under-fives (groups specifically targeted by the Millennium Development Groups), we conclude that it should be a priority to study this policy in more depth in future, to provide lessons for the region on its costs and effects.

### Remaining research questions

For the under-18 s policy, all of the process questions which this study aimed to answer remain unanswered. As a priority, the MoH and the NHIS should seek to document how the policy is operating, as well as addressing the wider questions listed below.

For the pregnant women’s free care policy, it is hoped that a future evaluation will focus on the following priority questions. These are of relevance not only to Ghana, but beyond, as countries search for effective mechanisms to extend universal health coverage

• What is the cost of the policy, and how is the cost profile changing over time?

• Who has benefited from it?

• How has utilisation responded for the various services (antenatal care, supervised deliveries, caesareans, postnatal care)?

• How have facilities been affected, and how have they reinvested additional resources (if any)?

• How have staff incentives been affected?

• What impact has the policy had on quality of care?

• How much variation in performance of the policy has there been between the regions, between the sectors (public, private and mission), and between levels of the health system?

• What has driven those variations in performance?

• What impact is the policy having on NHIS financial sustainability?

• How cost-effective is the policy as a whole?

## Conclusions

Ghana has to be congratulated for its persistence in trying to address financial barriers. Maternal utilisation and health indicators are improving over time, though supervised delivery remains relatively low and hard to shift. However, many themes from previous evaluations have recurred in this study – particularly, the difficulties of getting timely funding to facilities, of controlling charging of patients, and of reaching the poorest. These themes are consistent with other studies in the region, and suggest that shifting funding to the NHIS has not addressed some of the broader systemic issues raised in relation to stand-alone exemption policies. Countries such as Cote d’Ivoire, Mali and Senegal which are considering how to integrate exemptions into their national health insurance systems should reflect on the challenges which this may bring. Given that both policies are supported by general NHIS revenues, their longer term sustainability is also linked to the good management of that scheme. The wider concerns about raising quality of care, and ensuring that all supply-side and demand-side elements are in place to make the policy effective will take a longer term and bigger commitment. Given the absence of information and insights into the under-18 s policy, that would benefit from an urgent review of implementation and effectiveness.

## Competing interests

SW, BG and VR have no competing interests to declare.

## Authors’ contributions

SW, BG and VR designed the research. BG led on the key informant interviews and contributed to the analysis of KIIs. SW led on the literature review, analysis of interviews and drafting. All authors have approved the final version of the article.
